# Understanding on CRISPR/Cas9 mediated cutting-edge approaches for cancer therapeutics

**DOI:** 10.1007/s12672-022-00509-x

**Published:** 2022-06-08

**Authors:** Rudrarup Bhattacharjee, Lopamudra Das Roy, Amarendranath Choudhury

**Affiliations:** 1 Adelaide Medical School, Faculty of Health and Medical Sciences, The University of Adelaide, Adelaide, SA 5000 Australia; 2 Breast Cancer Hub, Concord, NC 28027 USA; 3 Department of Zoology, Patharkandi College, Patharkandi, Karimganj, 788724 Assam India

**Keywords:** CRISPR, Biotechnology, Cancer therapy, Genome editing

## Abstract

The research focus on CRISPR/Cas9 has gained substantial concentration since the discovery of ‘an unusual repeat sequence’ reported by Ishino et al. (J Bacteriol 169:5429–5433, 1987) and the journey comprises the recent Nobel Prize award (2020), conferred to Emmanuelle Charpentier and Jennifer Doudna. Cumulatively, the CRISPR has a short, compact, and most discussed success of its application in becoming one of the most versatile and paradigm shifting technologies of Biological Research. Today, the CRISPR/Cas9 genome editing system is almost ubiquitously utilized in many facets of biological research where its tremendous gene manipulation capability has been harnessed to create miracles. From 2012, the CRISPR/Cas 9 system has been showcased in almost 15,000 research articles in the PubMed database, till date. Backed by some strong molecular evidence, the CRISPR system has been utilized in a few clinical trials targeted towards various pathologies. While the area covered by CRISPR is cosmic, this review will focus mostly on the utilization of CRISPR/Cas9 technology in the field of cancer therapy.

## Introduction

The discovery of CRISPR (Clustered Regularly Interspaced Short Palindromic Repeat) system, although indirect, dates back to 1987, while Ishino and group found five 29 nucleotide long homologous sequences arranged as direct repeats having spacer of 32 nucleotides in the 3′ end of the gene *iap*, they were studying for *Escherichia coli *(*E. coli*) phosphate metabolism [1]. Around 6 years later, Mojica et al. while studying the salinity tolerance by the archaea *Haloferax mediterranei *(*H. mediterranei*), found some 30bp long DNA sequences having repeats at regular intervals. Upon further investigation showed 14 near-perfect conserved repeats which had degenerated copies flanking one end of the segment [2]. In the year 2000, many other clustered repeats of DNA were discovered in different archaea and bacteria, which were then termed as Short Regularly Spaced Repeats (SRSR) [3]. In the year 2002, the first article was published where the term ‘CRISPR’ was utilized for the first time. In-silico studies identified four CRISPR associated genes (Cas) present adjacent to the CRISPR loci. These genes were absent from prokaryotes, negative for CRISPR elements [4]. In the year 2005, three independent research groups identified similarity in sequences between spacer regions of CRISPR with regions of bacteriophages and plasmids, where, for the first time, it was defined as an immune system for bacteria to evade their foreign attackers [5–7]. Around the same time, an essential motif which imparts CRISPR its immunity function has been mentioned in some strains of *Streptococcus* bacteria. These essential motifs were subsequently named as Protospacer Adjacent Motif (PAM) [5–8]. In the year 2007, Barrangou et al. utilized a phage-host model system to demonstrate the immunity providing capability of CRISPR/Cas system. They utilized a phage-sensitive strain of *Streptococcus thermophilus* along with two virulent bacteriophages, then generated several phage resistant mutant bacteria by challenging the wild-type strain with different phages. It has been found that new spacers were incorporated into the CRISPR loci imparting resistance to the exposed phage. It was also shown that when those acquired spacers were removed, the bacteria lose its resistant to the phage, thereby highlighting the importance of spacer acquisition as an essential step for CRISPR immunity [9]. The following year marked the elucidation of the molecular mechanism behind CRISPR based immunity in bacteria. Brouns et al. tagged the Cas proteins and performed affinity purification leading to identification of a complex of five Cas proteins viz., CasA, CasB, CasC, CasD and CasE. The complex was termed as Cascade (CRISPR-associated complex for antiviral defence) and was recovered from *E. coli* lysates. The guide RNA is a very essential component of this Cascade system, made up of Cas9-crRNA (CRISPR RNA) and tracrRNA (trans activating CRISPR RNA), which ultimately guides the Cas9 enzyme to produce double stranded breaks in the target DNA thereby establishing the bacterial defence system [10, 11]. The guide RNA system has been further modified to fuse the crRNA and tracrRNA to formulate a single guide RNA (sgRNA), making it more convenient to use in gene editing applications [44].

In the year 2012, in-vitro experiments established the RNA-guided endonuclease mechanism of CRISPR/Cas9 complex [11] and it has been also deciphered that Cas9 produces the cuts in DNA [12]. These studies, for the first time, highlighted a great potential of the CRISPR/Cas9 system as a gene editing tool having a precise target identification and cleavage mechanism. The year 2013 marked the establishment of CRISPR/Cas9 system as a tool for eukaryotic genome editing, where targeted genome editing of mouse and human cells were demonstrated [13]. Another study utilized a modified CRISPR system called CRISPR-on/CRISPR-a (Cas9 nuclease dead and fused with transcription activator and single guide RNA (sgRNA)) to activate certain reporter genes in the human as well as mouse cells [14]. In the same year, another group utilized EGFP (enhanced green fluorescent protein) tagged endonuclease-deficient Cas9 along with sgRNA for high resolution imaging of repetitive elements in telomeres of chromosome as well as coding genes of a living cell [15]. Another exploration led some researchers towards utilizing Cas9 and sgRNA injection into mouse zygote that rescued a dominant mutation in *Crygc* gene which causes cataract in mice, demonstrating the efficacy of zygote level gene editing using CRISPR [16].

In 2014, Wang et al. explored the benefits of CRISPR/Cas9 system over traditional functional screening methods, where they studied the phenotypes requiring complete loss of function of gene. They also got benefited from lower false negatives in case of large-scale screen along with lower off-target effects of the screening method [17]. During 2014, the ability to utilize CRISPR/Cas9 system in primates was also demonstrated. Niu et al. produced a genetically modified monkey by targeting several genes in the embryo (at one cell stage) of the monkey, where they injected Cas9 mRNA and sgRNA. The system successfully targeted the genes *Ppar-γ and Rag1* with minimal off-target effects [18]. Genetic editing of human embryos using CRISPR/Cas9 system was first published in the year 2015. Liang et al. used the CRISPR system to target β-globin gene in tripronuclear zygotes, where they achieved effective cleavage of the gene, however, off-target cleavage was also observed and rate of homology directed repair (HDR) was low and subsequent interference by delta-globin DNA region with HDR oligos created unwanted mutations as well [19]. Another group utilized *Streptococcus pyogenes *(*S. pyogenes*) derived Cas9 (spCas9) system to model human liver disease in mice by targeting the *Pten* gene, which mediates non-alcoholic steatohepatitis (NASH). The generated mouse model showed the NASH characteristics which were comparable to the models generated using Cre-loxP system earlier [17].

In the year 2016, Liu et al. utilized CRISPR/Cas9 system to edit DNA methylation patterns in cells. They fused catalytically inactive Cas9 with the enzymes Tet1 (teneleven translocation methylcytosine dioxygenase 1) or Dnmt3a [DNA (cytosine-5)-methyltransferase 3 alpha] to achieve the methylation editing. The fusion protein targeting showed that it can activate or silence an endogenous reporter thus establishing its capability [20]. CAR (chimeric antigen receptor)-T cells have been game changer in cancer therapeutic field, and in 2016, it has been observed that Programmed cell death protein 1 (PD-1) blockage through antibody or knocking down the *PDCD1* gene that encodes PD-1, enhances the antitumour activity of CAR-T cells [21, 22]. The year 2016 also marked the improvement of T cell therapeutics via CAR-T cells where CRISPR/Cas9 system was utilized to reduce alloreactivity. The researchers made the endogenous T-Cells deficient of T-Cell receptor (TCR) along with HLA (human leukocyte antigen) class 1 which resulted in prevention of graft rejection by host system [23]. Another breakthrough happened in 2016 when Liu group at Harvard University and Kondo group at Kobe University, independently reported the development of a modified CRISPR editing technique known as BASE editing [24, 25]. The base editing technology do not require any external DNA template and using the partially inactive Ca9 nickase fused to a deaminase, the system can induce a permanent and precise single nucleotide change in the target DNA sequence without causing double stranded break [24, 25]. Within a short span of its development, the technology has been highly utilized in variety of preclinical studies in disease like progeria and sickle-cell anemia [26, 27]. In 2017, Rupp et al. utilized CRISPR/Cas9 to disrupt the PD1 gene and found marked improvement in CAR-T cell anti-tumour effect, both in-vitro and in-vivo [28]*.* The year 2017 also witnessed the use of type VI CRISPR system for editing RNA. Using the type VI Cas13 RNAse, researchers engineered REPAIR (RNA Editing for Programmable A to I Replacement) system which was capable of editing full length RNA transcripts which had pathogenic mutations [29].

CRISPR/Cas13 system has been utilized by researchers to develop a viral diagnostic technique, first published in 2018. They utilized a method called SHERLOCK (Specific High-sensitivity Enzymatic Reporter un-LOCKing) which amplifies RNA and then the amplified RNA is fused to Cas13a, its guide RNA along with a fluorescent reporter and quencher. On successfully finding the target sequence, Cas13a cleaves the reporter, thereby activating the fluorescent signal. This technique has been utilized to detect Dengue and Zika Virus presence in patient samples [30]. In 2019, Liu group at Harvard University developed another breakthrough CRISPR technology know, as PRIME editing. This technique utilizes a catalytically inactivated Cas9 enzyme fused to a reverse transcriptase guided by the prime editing guide RNA to cause small deletions or insertions in the target site, while still having the full capacity to introduce single nucleotide changes [31]. Expanding on CRISPR/Cas13a, Gao et al, in 2020 used a nuclear factor κB (NF-κB) driven gRNA expression for knocking down four endogenous oncogenes, viz., TERT, EZH2, and RelA in cell lines and in a mouse model where Hepa-1 -6 and WEHI-3 were xenografted. These CRISPR/Cas13a based approach showed significant reduction in tumour growth in both cell lines and the mouse model, further establishing the clinical utility of CRISPR/Cas13a method [32].

The year 2019 was a remarkable year in CRISPR history, where for the first time, CRISPR associated Phase 1 clinical trial in humans has been started in the US. The results of this trial were published earlier in 2020, where CAR-T cells showed promising potential towards cancer treatment [21]. The year also marked the first study that used LASER ART and CRISPR Treatment to eliminate HIV-1 in a humanized mouse model [33]. At the same time, Strecker et al. identified a new CRISPR gene editing system, where they engineered ShCAST [CRISPR-associated transposase from cyanobacteria *Scytonema hofmanni *(*S. hofmanni*)] which contained Tn7-like transposable element along with Cas12k (V-K CRISPR effector). The system utilizes DNA transposition guided by RNA which was used for DNA integration in *E. coli* genome at target sites [34]. Many cancerous growths contain cells with exclusive fusion oncogene (FO), which serve as a kind of marker for those cells. Martinez-Lage et al. in 2020, explored the therapeutic targeting of FO containing cancers by a CRISPR technique targeting introns of the genes involved in the FO formation. They showed that such intron targeting of genes for transcription factors or tyrosine kinases, required for the FO cancer cells to survive, showed great potential in mouse models where reduced mortality and less tumour burden was observed [35].

**Fig. 1:**
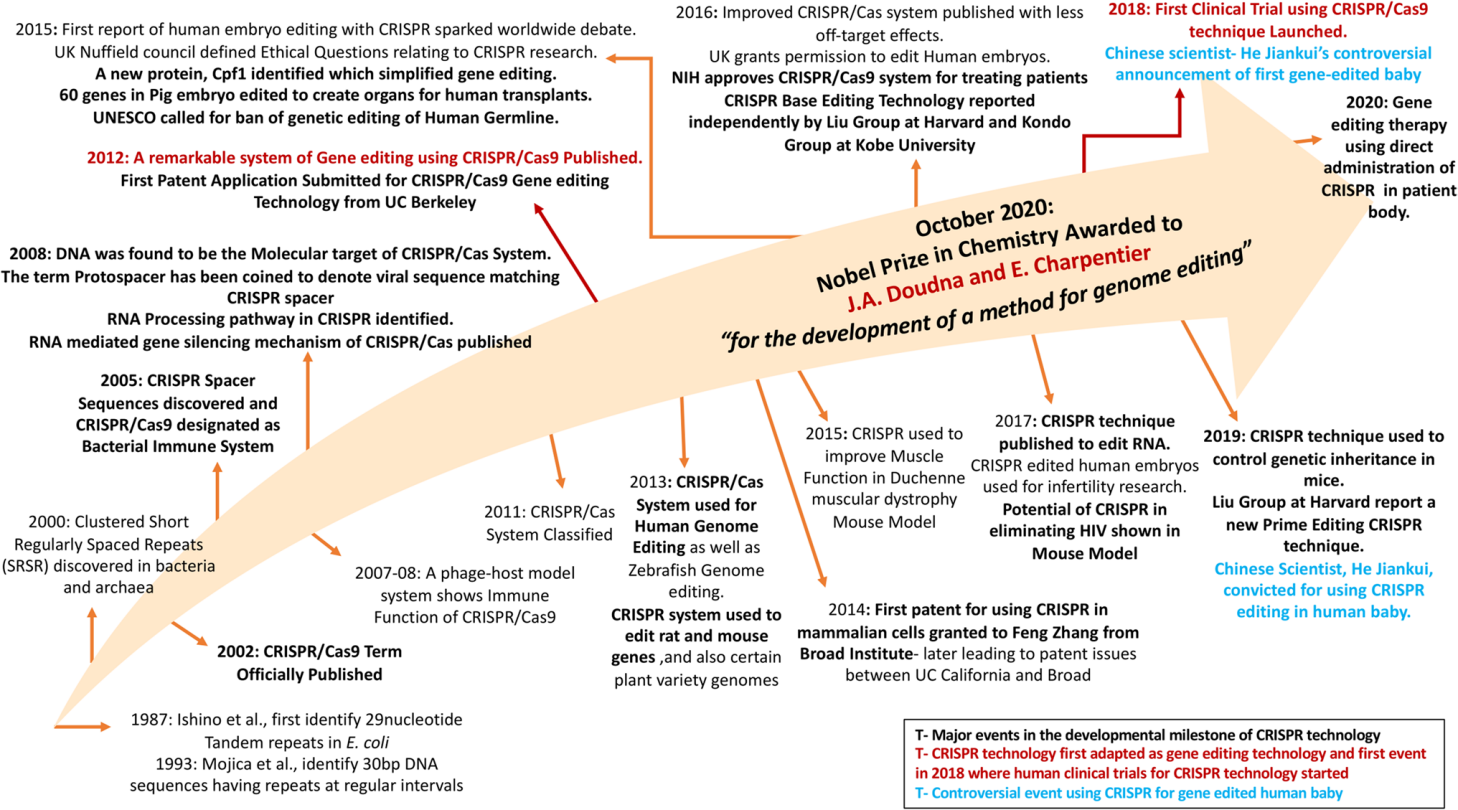
Timeline of CRISPR research

In 2020, outbreak of Coronavirus Disease-2019 (COVID-19) caused a global pandemic, and at the same time necessitated the evolution of a fast and accurate system of diagnosis to detect the virus in patients. The CRISPR/Cas13a based system was utilized to develop a detection and quantitation assay for the coronavirus. The approach derives it novelty in detecting the RNA directly without any extra manipulation steps like amplification. A single guide RNA has been utilized to detect the viral RNA combined with a fluorescent signal, which can be detected using a mobile camera. Available in its current form in medRXiV, the system shows high potential as being an efficient and fast system for detecting coronavirus in patients [36]. The year 2020 witnessed the prestigious Nobel Prize in Chemistry being awarded to Jennifer A. Doudna and Emmanuelle Charpentier “*for the development of a method for genome editing*” [37], which revolutionized the gene editing world in a never before way and continues to evolve as a more versatile system day by day. A historic perspective highlighting the significant milestones of CRISPR research has been depicted in Fig. 1 of this manuscript.

## CRISPR/Cas9: structural overview

Identified as a sophisticated adaptive immunity of bacteria, the CRISPR/Cas9 system has revolutionized the genetic editing field. These are organizationally unique short, repeated DNA sequences having a partially palindromic nature that make bacteria immune to the invading viruses [9]. When a bacterium is invaded by a virus or any other bacteria, short fragments of the genetic element from those invaders get integrated into the CRISPR spacer array of the host bacteria serving as new spacers specific to the identity of the invading pathogen [38]. The CRISPR-array then gets transcribed and processed via endonucleolytic cleavage producing mature CRISPR-RNAs (crRNAs). The 5′ end of this crRNA contains the spacer (foreign material from invader), while its 3′ end contains a repeat sequence of CRISPR. When the invader attacks again, complementary binding between the spacer element in the crRNA and the invader genetic element (called protospacer) triggers a cascade of events leading to destruction of invader DNA or RNA via Cas nuclease systems, thereby providing immunity to the host bacteria [39–41]. The assembly of a mature crRNA with the Cas proteins leads to the formation of an ‘Effector Complex’ which actively searches for the invader and on spacer sequence match, causes degradation of the invading genetic element [42–44]. This whole process, however, relies a lot for its specificity upon a short motif of nucleotides (~3) known as Protospacer Adjacent Motif (PAM), which mediates the most specific cleavage of the target genetic element [8, 45–48]. Based on the different effector systems and proteins involved, the CRISPR-Cas systems have been classified into two broad classes i.e., Class1 and Class 2, having different sub-groups associated with them. A detailed classification has been given in Table 1 along with citations of the relevant studies. However, among all the described systems, the type 2 systems have been utilized for genetic engineering purposes. The major advantage of type 2 system relies on its effector enzyme i.e., Cas9 and its precise guide RNA system for locating the target DNA.

**Table 1:** Different CRISPR-Cas systems with their effector molecules [54, 55]

Class	Type	Sub-type	Feature	Effector molecule	Organism	References
Class 1	Type-I		Multisubunit effector complex, signature gene *cas3*	Cascade	*Escherichia coli*	Brouns et al. [10]
	Type III	III-A	Csm effector module, signature cas10 with Palm Domain; targets DNA	Cas10-Csm	*Staphylococcus epidermidis*	Marraffini and Sontheimer [39]
		III-B	Cmr5 effector, targets mostly RNA	Cmr	*Pyrococcus furiosus*	Hale et al. [40]
	Type-IV (Putative)		multisubunit crRNA–effector complex, Csf1 signature gene with Cas5 and Cas7	Csf1-Cas5, Cas7	*Acidithiobacillus ferrooxidans*	Makarova et al. [55]
Class 2	Type-II		crRNA and tracrRNA, single effector molecule	Cas9	*Streptococcus thermophilus, Streptococcus pyogenes*	Bolotin et al. [8]; Barrangou et al. [9]; Sapranauskas et al. (2011); Gasiunas et al. [11]; Deltcheva et al. [93]; Jinek et al. [12]; Cong et al. [13]; Mali et al [60]
	Type-V		Single effector molecule, guided by Single RNA	Cpf1	*Francisella novicida*	Zetsche et al. [94]

Whether in natural or engineered CRISPR/Cas systems, Cas9 effector complexes are very efficient in targeting the precise DNA locus. The Cas9 enzyme derives is function on the basis of six functional domains, viz., REC I, REC II, Bridging Helix, PAM Interaction Domain, HNH domain and RuvC domain [49, 50]. The REC I domain mediates binding of the enzyme to the guide RNA (crRNA-tracrRNA or sgRNA), while the function of REC II domain still remains to be elucidated. The Bridging Helix is rich in Arginine residues and helps initiate target DNA cleavage upon successful binding. The PAM domain imparts specificity to bind to the PAM sequence and consequently mediates target DNA binding with high specificity [49–52]. The RuvC and HNH domains serve as the cleavage domains and share high homology with other HNH and RuvC domains of different proteins. These are the primary nuclease domains and mediate the target DNA cleavage, where HNH cleaves the Target strand of the DNA while RuvC causes cut in the nontarget strand of the DNA [49, 50].

Owing to the high specificity, simplicity of use, quick and easy design combined with high efficiency and minimal off-target effects, the CRISPR/Cas system, since its first genetic engineering potential report [12], has now evolved as an unparalleled genome manipulation tool covering a wide variety of organisms [53]. It is unique to the other genetic editors like Zinc finger Motifs (ZFM) or Transcription activator-like effector nucleases (TALEN), owing to its RNA mediated target recognition, thereby eliminating the need for repeated protein engineering to identify diverse DNA targets, making it easily scalable and broader in usability among the diverse fields of genetic engineering.

## CRISPR/Cas9 mechanism of action

**Fig. 2:**
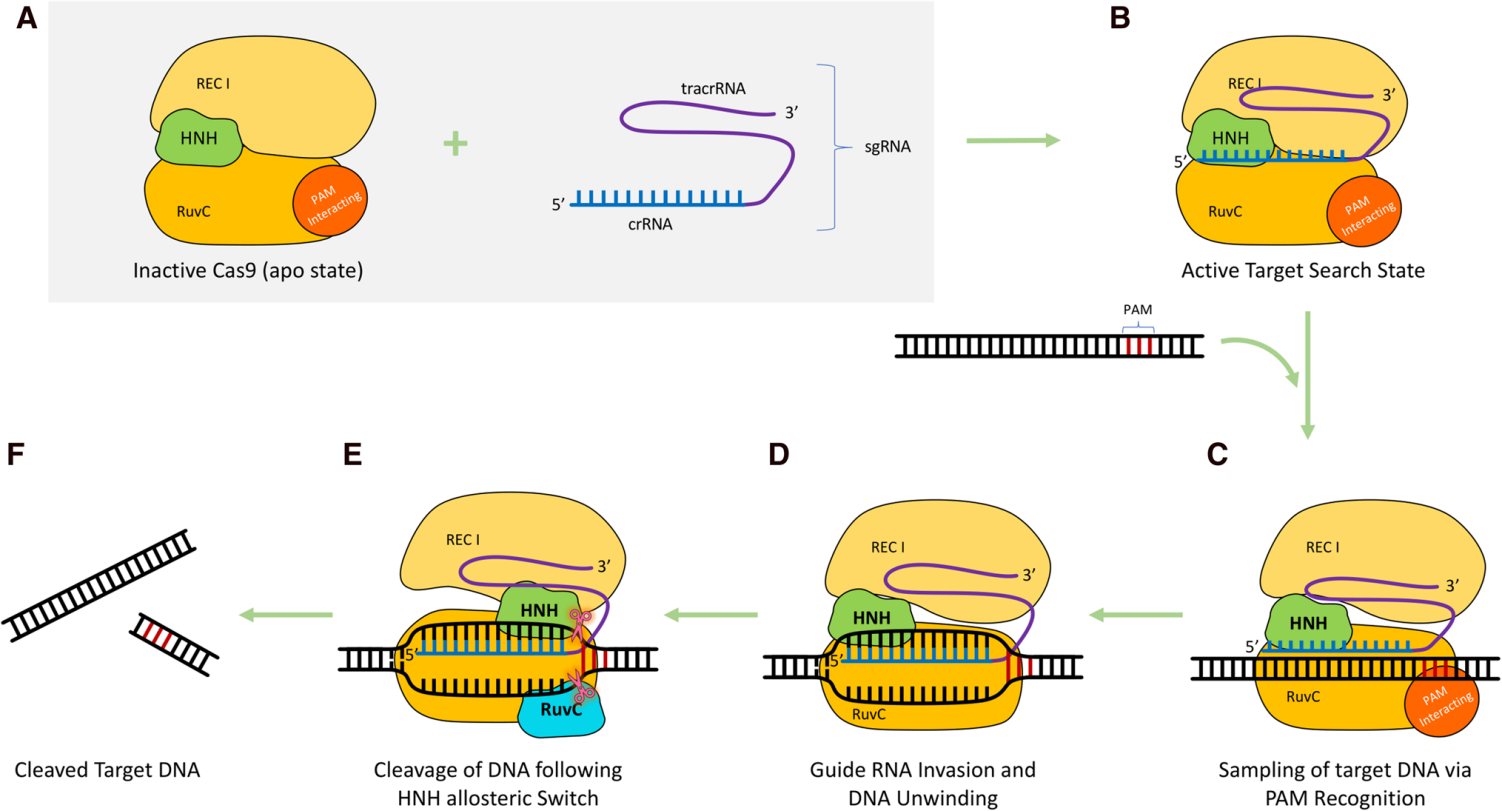
Molecular mechanism of CRISPR/Cas9 action

The Cas9 enzyme remains in an apo-state (inactive) (Fig. 2A) until being bound by a guide RNA (gRNA) complex, either the natural crRNA-tracrRNA or an engineered single guide RNA (sgRNA), which alters the enzyme configuration towards a target DNA surveillance state (Fig. 2B) [12, 49]. The 20-nucleotide region in the crRNA called the spacer region is crucial for imparting DNA target specificity through its seed sequence [37, 56, 57]. The seed region is of particular interest as mismatch in this region can impair or even completely revoke DNA binding, whereas high homology with this region has been found leading to many off-targets binding even with mismatches in other regions [58].

Following gRNA binding, the first conformational change occurs in the recognition (REC) domain of the Cas9 enzyme where the Hel-III region moves around 65Å towards the HNH Domain [48]. However, after binding of target DNA and PAM sequences, further conformational change remains minimal, which strengthens the idea that gRNA loading is the major regulator of Cas9 functional cascade [49]. The major interactions between Cas9 and the sgRNA takes place via the stem-loop 1 and repeat-antirepeat duplex of the enzyme. The stem-loop 2 and 3 are found to be dispensable parts which are not essential but may strengthen the active Cas9 effector complex formation imparting better catalytic efficiency. Biochemical studies have confirmed this notion and it has been further identified that deletion of stem-loop1 completely impairs the cleavage function of Cas9, making it the most important region for the enzymatic activity [13, 50, 59, 60]. Before making contact with target DNA, the seed sequence of gRNA and PAM interacting regions R1333 and R1335 undergo prepositioning to activate the Cas9 enzyme complex towards a competent DNA recognition state [48].

After gRNA binding and subsequent conformational change, the Cas9 is ready to search and bind the target DNA (Fig. 2C). The target recognition and binding occur via complementary base pairing between 20 nucleotide spacer sequence and the protospacer region in target DNA. However, this interaction also requires the presence of a conserved PAM sequence which helps distinguish between self and non-self-sequences [11, 12, 39]. The target DNA search by Cas9 starts by probing for a correct PAM sequence before it surveys the flanking regions, as demonstrated via certain single molecular experiments [52]. If the PAM mismatches, the Cas9 rapidly dissociates from the DNA and begins a new search. However, once the PAM is recognised, scanning through flanking regions takes place and subsequently local melting of the target DNA starts at the nucleation site near the PAM sequence [52, 61, 62]. This is followed by RNA invasion and subsequent formation of RNA-DNA heteroduplex (Fig. 2D) [63]. This process is mediated by the ssDNA-bound structure model, where the spacer sequence of the gRNA gets hybridized to the target DNA sequence via a 20 nucleotide Watson-Crick base pairing, thereby forming a heteroduplex [50]. Further studies of this structure identified that the Cas9 identifies the geometry of the heteroduplex in a sequence independent way and the PAM-duplex structure maintains the pseudo-A conformation of the RNA-DNA hybrid [51].

Studies show that recognition of PAM leads to destabilization of the adjacent sequences locally and the interactions between a phosphate lock-loop and +1 Phosphate contributes to the melting of DNA duplex and subsequent stabilization of RNA-DNA heteroduplex [12, 51, 52]. Structural studies show that Cas9 association with target DNA causes a bend in the DNA duplex shifting it from 180° to ~ 150°, in the bound region [48]. Such a DNA bending by Cas9 (redolent of RNA Polymerase action during transcription) may help strand separation and prevent re-hybridization for an efficient cleavage of the target DNA [64, 65].

After successful completion of all the above events, the Cas9 enzyme proceeds towards DNA cleavage using its two nuclease domains, viz., RuvC domain and the HNH domain [48, 52]. Each of the domains cut one strand of the dsDNA at a specific site 3bp adjacent to the PAM sequence (Fig. 2E) thereby producing double stranded breaks (DSB) with blunt ends (Fig. 2F) [11, 12].

One of the much-raised questions about such DNA editing is the off-target events that might happen due to promiscuous DNA binding by the CRISPR/Cas9 complex, having seed sequence similarity as little as 5 nucleotides [66, 67]. However, through extensive experimentation, it has been proven that the cleavage event is decoupled from just the binding and mere binding itself does not cause cleavage of the bound DNA [66, 68]. It has been further established that to have a successful cleavage, a conformational change has to occur in the activated enzyme complex stabilized by interaction of the gRNA with the 14–17th base pair region of the protospacer [69]. While this may not be a system for complete ablation of off-target effects, this indeed ensures that off-target events, if any, may occur minimally [52].

After the stable establishment of gRNA and target DNA base pairing (via PAM recognition), a conformational change within the Cas9 triggers RNA invasion and binding beyond seed region in the target strand of the dsDNA, thereby un-constraining the non-seed regions. The base pairing then continues towards the 5′ end of the guide sequence inducing further conformational changes within the Cas9 enzyme making it finally active [48, 50–52, 63, 69, 70]. The HNH domain of the enzyme then gets activated for target strand cleavage causing the loop linkers to undergo a further conformational change facilitating the concerted cleavage of non-target strand by RuvC [52, 70]. On completion of the DNA cleavage, Cas9 remains bound to the DNA until displaced by certain cellular factors for recycling [52].

## Application of CRISPR/Cas9 in cancer

Since the discovery of CRISPR-Cas9 technology, new applications are emerging to study the role in cancer development and progression, providing ideas into how to prevent or treat refractory cancers. Given the versatility of this technique, it has been applied into many biological systems like primary mouse and human cells, organoids, different cancer cell lines up to generating various genetically engineered mouse models [71].

Majority of the chemotherapy for cancer requires and continued dosage, which aggravates the treatment induced toxicity as well as the cost for the patient, however, it also causes severe deterioration in the quality of life of the patient. The CRISPR technology holds the potential to permanently disrupt the genes required for tumour cell survival thereby ablating the need of repeated chemotherapy administration, which ultimately may improve treatment efficacy while requiring less treatment visits, reducing the cost. However, achieving an effective treatment will require a significantly high gene editing efficiency to get the therapeutic benefit [72, 73]. A recent study by Rosenblum et al. reports that CRISPR-Lipid nanoparticles (cLNPs), when targeted against *PLK1* (sgPLK1-cLNP) in fast-growing glioblastoma, was able to achieve ~ 70% in-vivo gene editing (using single intraperitoneal inoculation) leading to cellular apoptosis in the tumour, which, in turn, caused up-to ~ 50% reduction in tumour progression while increasing the survival rate by 30% at the same time. Similarly, when sgPLK1-cLNPs (coated with anti-EGFR) were delivered intraperitoneally, they got selectively taken up by ovarian tumours leading to almost 80% in-vivo gene editing. This led to inhibition of tumour growth while increasing rate of survival (~ 80%). This effort underpins new possibilities where genome editing can be applied to treatment regimens, thereby advancing CRISPR technology towards the clinic [74]. Another advancement highlights the use of CRISPR/Cas9 technology in combination with CAR-T cell technology showed better safety and therapeutic efficacy for the CAR-T cells, which, in-turn, showed promising results against tumours in animal models as well as *in-vitro situations* [75]. Additionally, the T cells generated with CRISP, by making them deficient in both TCR and HLA class I, showed much reduced alloreactivity, while also avoiding graft-versus-host disease [23]. Further to this, a study showed that by disrupting *PD1* gene also, triple deficient CAR-T cells can be generated, which showed a significant increment in their in-vivo antitumor potential [76]. Therefore, combing these two revolutionary techniques is giving rise to a new era of oncotherapy using CAR-T cells.

With rapid technological advancement, CRISPR is moving rapidly towards clinical use, as an effort to get new scientific tools from ‘bench to bedside’. Majority of recent studies indicated towards an improvement in cancer therapy in terms of immunological interventions where CRISPR edited adaptive cells transfer plays a crucial part. Apart from that, novel DNA and RNA detection regimens using CRISPR-tools also show promising implications in tumour genotyping and diagnosis [77].

## CRISPR/Cas9 technology in cell-based therapeutics

Lu et al. in their 2020 Nature Medicine paper explored the safety and feasibility of *PD-1* targeting CRISPR edited T-cells in therapeutic management of advanced non-small-cell lung cancer (NSCLC) patients. Interestingly, the approach had very minimal off-target effect and did not cause any treatment induced adverse event, thereby making a strong case for clinical utility of CRISPR technology [78]. These results also support the findings of two recent clinical trials examining clinical feasibility and safety of CRISPR based cell therapies. In one of them, Xu et al. engineered *CCR5*-ablated hematopoietic stem & progenitor cells (HSPC) by CRISPR/Cas9 gene editing and transplanted them into a HIV-1 positive patient, also having acute lymphoblastic leukemia (ALL). They found that ALL was in full remission in the patient along with complete donor chimerism, while the *CCR5* ablated cells persisted in the patient for more than 19 months. It becomes further exciting as there was no gene engineering related adverse event noted in the patient. This study furnishes an excellent example of long-term allogenic stem-cell transplantation with CD34+ cells (having CRISPR edited *CCR5*) where only < 8% gene disruption noted in circulating bone marrow cells. Interestingly, they study did not find any off-target effects of the CRISPR edits in the patient [79]. In another study, Stadmauer et al. isolated T-cells from two progressive stubborn myeloma patients and one advanced sarcoma patient and used CRISPR/Cas9 where they disrupt the *TRAC, TRBC*, and *PDCD1* genes and inserted the *NY–ESO-1* transgene (which recognize tumours) to get an improved antitumor immunity [24]. The engineered CAR-T cells, when injected to the patients, remained stable for almost 9 months, while also showing promising tolerability and no clinical toxicity in the patients. These encouraging observations pave the way for future trials to study CRISPR-engineered cancer immunotherapies, a step towards revolutionizing the treatment scenario in patients with advanced cancer [21]. However, experience with more patients given infusions with higher editing efficiencies and longer observation after infusion will be required to fully assess the safety of this approach.

The CRISPR/Cas9 system emerged as a versatile yet very robust gene modification toolset, with immense potential in the cancer therapeutics. It’s ability to discover novel targets and subsequent application makes it excellent therapeutic candidate. However, CRISPR do come with its own unique challenges and limitations and therefore, further research is needed to regulate the layout and determine the potential with clinical responses to understand the safety, efficiency and feasibility on large scale. Having said that, the CRISPR/Cas9 technology is undoubtedly moving blazing fast, emerging as a promising therapeutic means for Cancer treatment.

## Current progress and promises in cancer therapy through CRISPR/Cas9

While being extensively used in research, the potential of CRISPR has also been tested towards clinical trials for a few cancer pathologies. The first in-human phase-1 trial, started in 2019 by Prof Carl June’s group at University of Pennsylvania, reported some interesting findings during early 2020. They utilized a multiplex editing strategy using CRISPR/Cas9 to edit T cells in three refractory cancer patients. Firstly, they enhanced the expression of a cancer cell specific T-cell receptor (TCR) called *NY-ESO-1*, and then to remove interference in this receptor activity, they deleted two endogenous TCR chains viz, TCRα (*TRAC*) and TCRβ (*TRBC*). The deletion of these two genes also reduced mispairing of TCR. They also deleted another gene called programmed cell death protein 1 (*PD-1/PDCD1*) to enhance antitumor efficiency. Substantial engraftment was achieved for those engineered T-Cells having three genomic loci edited. Despite the fact, there were occasions of chromosomal translocations, which although decreased over time, pose a significant question of off-target effects. The edited T-cells could survive up to 9 months in the patient body showing minimal immunogenicity against themselves [21]. However, of all the cells used in the therapy, only 10% had all the four CRISPR edits and all the 3 participants who received these cells had some off-target effects, although these effects did not cause any sort of malignancy. The effect on the patients was small and the cancerous growth in two patients, being stopped the growth for some time, did relapse after some time. Still in the very early phase, even the small success against solid tumours has been remarkable. The authors, however, advocate that the after effects needs to be evaluated continuously and the patients need to be watched regularly for several years to ascertain the real-world scenario of CRISPR feasibility [21]. Although not an absolute success, this first trial shows the possibility of using CRISPR in diverse forms towards cancer therapeutics, of course with more advancement of the system to minimize the potential limitations.

Apart from this published study, there are few other CRISPR associated trials currently ongoing in terms of cancer treatment, while many others started and ended without reporting any solid data. A detailed overview of all the current trials (actively updating information and revised time to time) for cancer which involves CRISPR/Cas strategy are tabulated below in Table 2.

**Table 2:** Ongoing clinical studies on therapeutic potency of CRISPR/Cas9 in cancer ( Source: Clinicaltrials.gov., PubMed)

Sl. No.	Title	Condition	Treatment technique	Organization
1	A phase 1 dose escalation and cohort expansion study of the safety and efficacy of allogeneic CRISPR-Cas9-engineered T cells (CTX110) in subjects with relapsed or refractory B-cell malignancies (CARBON)	B-cell lymphoma, non-hodgkin lymphoma,	Allogenic T-cells modified genetically (in-vivo) using CRISPR to edit *CTX110* in CD19-directed T-cell immunotherapy	CRISPR Therapeutics AG
2	Phase I Study to Evaluate Treatment of CRISPR-Cas9 Mediated PD-1 and TCR Gene-knocked Out Chimeric Antigen Receptor (CAR) T Cells in Patients with Mesothelin Positive Multiple Solid Tumours	Adult Solid Tumour	Infusion of anti-mesothelin CAR-T cells On Day 0	Chinese PLA General Hospital
3	A Phase I/II trial in patients with metastatic gastrointestinal epithelial cancer administering tumour-infiltrating lymphocytes in which the gene encoding CISH was inactivated using the CRISPR/Cas9 system	Cancers of colon, stomach, gastrointestinal tract, esophagus, gallbladder, pancreas	Drug: cyclophosphamide	Intima Bioscience, Inc./Masonic Cancer Center, University of Minnesota
			Drug: fludarabine	
			Biological: tumour-infiltrating lymphocytes (TIL)	
			Drug: aldesleukin	
			Other name: interleukin-2, IL-2	
4	A phase 1, open-label, multicenter, dose escalation and cohort expansion study of the safety and efficacy of anti-CD70 allogeneic CRISPR-Cas9-engineered T cells (CTX130) in subjects with relapsed or refractory T or B cell malignancies	T cell lymphoma	*CTX130* CD70-directed T-cell immunotherapy comprises allogeneic T cells genetically modified ex vivo using CRISPR-Cas9 gene editing components	CRISPR Therapeutics AG
5	CRISPR (HPK1) edited CD19-specific CAR-T cells (XYF19 CAR-T cells) for CD19 + leukemia or lymphoma	Leukemia, lymphocytic acute (ALL) in relapse	CD19 specifying engineered T-Cells (autologous) transduced with lentivral vector followed by electroporation of guide RNA for CRISPR disrupting endogenous *HPK1* gene, and then cells administered using IV injection. Two drugs, viz., Cyclophosphamide and Fludarabine were also used	Xijing Hospital /Xi'An Yufan Biotechnology Co., Ltd.
		Leukemia lymphocytic Acute (all) refractory		
		Lymphoma, B-Cell		
		CD19 positive		
6	A phase 1 dose escalation and cohort expansion study of the safety and efficacy of allogeneic CRISPR-Cas9-engineered T cells (CTX130) in subjects with advanced, relapsed or refractory renal cell carcinoma with clear cell differentiation	Renal cell carcinoma	Allogenic T-cells modified genetically (ex-vivo) using CRISPR to edit *CTX130* in CD70-directed T-cell immunotherapy	CRISPR Therapeutics AG
7	TACE combined with CRISPR/Cas knockout of PD-1 engineered T cell in advanced hepatocellular carcinoma	Advanced hepatocellular carcinoma	The patients having Transcatheter arterial chemoembolization (TACE) operation planned, are going to receive 3 or more cycles of *PD-1* knockout engineered T cells infusion by with a 4-weeks interval. CRISPR engineered *PD1* knockout T-cells to be given for 3 or more cycles to the patients using a percutaneous fine needle liver puncture at 4 weeks intervals	Central South University
8	Phase 1of CRISPR-CAR genome edited T cells (PBLTT52CAR19) in relapsed/refractory B cell acute lymphoblastic leukaemia	B-cell acute lymphoblastic leukemia	Single infusion of CRISPR engineered allogenic T cells transduced with a self-inactivating (SIN) lentiviral vector in up to 10 subjects (age from 6 months to 18 years) with a hope to get remission of relapsed or refractory B-cell acute lymphoblastic leukaemia (B-ALL)	Great Ormond Street Hospital for Children NHS Foundation Trust/University College, London
9	A phase 1, multicenter, open-label study of CB-010, a CRISPR-edited allogeneic anti-CD19 CAR-T cell therapy in patients with relapsed/refractory B cell non-hodgkin lymphoma (ANTLER)	B cell non-Hodgkin’s lymphoma, relapsed and refractory	3 + 3 study design CB10A clinical trial consisting of three dose levels. A sequential assignment model to be followed for the study	Caribou Biosciences, Inc.

## Limitations in CRISPR/Cas9 mediated therapy

Although having the unique gene targeting potential, CRISPR/Cas9 suffers from a major limitation of off-target effects (OTEs) at a rate of around ~ 50%, which makes it difficult to use widely for gene therapy [80]. However, many strategies are currently being utilized to address this problem and two most significant approaches include engineered Cas9 and a few different optimization strategies towards designing the CRISPR guides [81]. Another limitation in this technique arises from the requirement of a PAM sequence near the target site. Although PAM imparts specificity, but having a PAM at a correct position often limits the choice of targets. Having a shorter PAM sequence i.e., 5′NGG3′ (N is any nucleotide), makes the *Streptococcus pyogenes* derived Cas9 (spCas9) one of the most widely used systems, however, larger size of the spCas9 makes it difficult to package in to Adeno Associated Virus (AAV), which is the most commonly used delivery vehicle in gene therapy [82, 83].

Another limitation of CRISPR tech arises from the fact that the double stranded breaks (DSB) created by CRISPR system often leads to apoptosis of the cells rather than desired genetic edit [84]. In a study using human pluripotent stem cells (hPSCs), it has been found that DSBs created by CRISPR often behave as toxic entities which trigger p53 pathway and subsequently leads to apoptosis, creating further safety concerns regarding the technology [85]. These findings also raised a question that successful CRISPR edits may more likely happen in p53 deficient cells thereby increasing bias towards survival of oncogenic cells [80]. Apart from these observations, it has also been found that successful on-target edits are often accompanied by unintentional deletions of large size and/or complex rearrangements in the genome highlighting a major limitation of CRISPR technology that uses DSB mechanism of edit [86]. A new strategy using catalytically inactive Cas9 or dead Cas9 (dCas9) may provide some alternate therapeutic utility while avoiding the risks of DSB mediated mechanism [87]. These possibilities are still being explored, and although successful in many research scenario, clinical safety and efficacy of such systems are still to be thoroughly studied and identified.

Apart from the technical limitations, CRISPR/Cas9 based therapeutics also suffers from the problems of immunologic toxicity, just as traditional gene therapy approaches. In a study, Charlesworth and colleagues demonstrated the pre-existence of anti-Cas9 antibodies against the most commonly used spCas9 and saCas9 (*Staphylococcus aureus*) proteins in almost half of the human subjects studied [88]. Furthermore, the most commonly used delivery vehicle for CRISPR system, AAV and several Cas9 orthologs were subjected to binding test with MHC class I and class II in order to determine orthologs that can be used repeatedly for CRISPR therapy without promoting much immune-toxicity. None of the AAV serotypes were found to completely avoid immune system recognition, but, three Cas9 enzymes i.e., spCas9, saCas9 and cjCas9 (*Campylobacter jejuni* Cas9) showed higher efficiency of edits along with low immunogenicity in mice which were immunized against AAV and Cas9 [89]. However, in humans, pre-existing immune response towards spCas9 and saCas9 has been identified which left only cjCas9 as the sole option for the study cohort of these patients [80]. Another limitation arises from the fact that cjCas9 is less explored as compared to the other two Cas9 enzymes and it needs much more elaborate studies to define and explore its efficacy as well as safety in terms of clinical utility [90]. Further exploratory studies to identify other Cas9 orthologs is, therefore, highly warranted to get safe and effective Cas9 variants to be used in clinical settings. While engineered Cas9 does address the issues of PAM restriction [91] and many high fidelity Cas9 were developed to increase on-target edits while limiting the off-target effects [92], the drawbacks still exist and gets more pronounced because of the larger size of the CRISPR tools to be delivered in the target cell/tissue. Thus, more refined CRISPR system with minimal off-target effects and at a size allowing their packaging to the widely used delivery vehicles are some key thrust areas to be addressed in near future. These optimizations will get CRISPR closer to the clinics against onychopathologies.

## Conclusion and future prospective

Altering genetic material using CRISPR still remains a much debated as well as controversial factor in terms of human utilization. However, a few approvals provided by Regulatory Affairs Certification (RAC) and FDA lead to the opening of a few clinical trials for gene therapy using CRISPR, which came after a great deal of review, thorough considerations, and weightage of risk to benefit ratio. Current trials in Phase I/II, although very limited, are designed only for patients with severe diseases as cancer or other monogenic debilitating disease, which otherwise remain untreatable. The outcomes from these studies may provide a snapshot as of how safe these tools would be for the less severe cases and how soon they may be implemented as more and more risks associated with the technologies will get discovered and addressed. The biggest concern, however, remains as the fact that generalizing CRISPR/Cas9 for less debilitating diseases might expose the technology towards non-therapeutic alteration of human genome including embryonic editing for inserting certain aesthetic traits in the offspring. Such a fear has realized more attention and became a tangible problem after the media had a strong coverage of the ‘CRISPR edited Babies’. While this unethical utilization remains a concern, regulated utilization of CRISPR towards gene therapy has to be considered and made feasible for near future. The outcome of trials mentioned earlier in this article will largely dictate the future of CRISPR technology in the gene therapy field, as these are slated to unravel certain key questions about both safety and efficacy of the CRISPR/Cas9 technique in treating otherwise untreatable disorders. To summarize, there remains two very broad questions to be answered before widespread use of CRISPR, firstly, how CRISPR’s therapeutic future will be decided and who will regulate the authorizations? And secondly, what ethical boundaries should be drawn to get most out of the technology while preventing its misuse to get the best outcome of this revolutionary gene editing technique? With these two questions answered and the various limitations addressed, CRISPR holds the key to take human clinical therapeutics to a great new height where miracles would be possible in terms of disease management and care.

## Abbreviations

CRISPR: Clustered regularly interspaced short palindromic repeat
Cas9: CRISPR-associated protein 9
iap: Inhibitors of apoptosis
DNA: Deoxyribonucleic acid
SRSR: Short regularly spaced repeats
PAM: Protospacer adjacent motif
RNA: Ribonucleic acid
tracrRNA: Trans activating CRISPR RNA
crRNA: CRISPR RNA
sgRNA: Single guide RNA
EGFP: Enhanced green fluorescent protein
PPARγ: Peroxisome proliferators–activated receptor γ
Rag1: Recombination activating gene 1
HDR: Homology directed repair
spCas9: *Streptococcus pyogenes* (*S. pyogenes*) Derived Cas9
NASH: Non-alcoholic steatohepatitis
Pten: Phosphatase and TENsin homolog deleted on chromosome 10
Tet1: Teneleven translocation methylcytosine dioxygenase 1
Dnmt3a: DNA cytosine-5-methyltransferase 3 alpha
CAR: Chimeric antigen receptor
PD-1: Programmed cell death protein 1
HLA: Human leukocyte antigen
SHERLOCK: Specific High-sensitivity Enzymatic Reporter un-LOCKing
NF-κB: Nuclear factor κB
TERT: Telomerase reverse transcriptase
EZH2: Enhancer of zeste homolog 2
RelA: REL-associated protein
ShCAST: CRISPR-associated transposase from cyanobacteria *Scytonema hofmanni*
FO: Fusion oncogene
COVID-19: Coronavirus disease-2019
HNH: His-Asn-His
ZFN: Zinc finger motifs
TALEN: Transcription activator-like effector nucleases
DSB: Double stranded breaks
cLNPs: CRISPR-lipid nanoparticles
NSCLC: Non-small-cell lung cancer
HSPC: Hematopoietic stem & progenitor cells
TCR: T-cell receptor
OTEs: Off-target effects
AAV: Adeno associated virus
RAC: Regulatory affairs certification
FDA: Food and Drug Administration

## References

[1] Ishino Y, Shinagawa H, Makino K, Amemura M, Nakata A (1987). Nucleotide sequence of the iap gene, responsible for alkaline phosphatase isozyme conversion in *Escherichia coli*, and identification of the gene product. J Bacteriol.

[2] Mojica FJ, Juez G, Rodríguez-Valera F (1993). Transcription at different salinities of *Haloferax mediterranei* sequences adjacent to partially modified PstI sites. Mol Microbiol.

[3] Mojica FJ, Díez-Villaseñor C, Soria E, Juez G (2000). Biological significance of a family of regularly spaced repeats in the genomes of Archaea, Bacteria and mitochondria. Mol Microbiol.

[4] Jansen R, van Embden JDA, Gaastra W, Schouls LM (2002). Identification of genes that are associated with DNA repeats in prokaryotes. Mol Microbiol.

[5] Haft DH, Selengut J, Mongodin EF, Nelson KE (2005). A guild of 45 CRISPR-associated (Cas) protein families and multiple CRISPR/Cas subtypes exist in prokaryotic genomes. PLoS Comput Biol.

[6] Mojica FJM, Díez-Villaseñor C, García-Martínez J, Soria E (2005). Intervening sequences of regularly spaced prokaryotic repeats derive from foreign genetic elements. J Mol Evol.

[7] Pourcel C, Salvignol G, Vergnaud G (2005). CRISPR elements in Yersinia pestis acquire new repeats by preferential uptake of bacteriophage DNA, and provide additional tools for evolutionary studies. Microbiology.

[8] Bolotin A, Quinquis B, Sorokin A, Ehrlich SD (2005). Clustered regularly interspaced short palindrome repeats (CRISPRs) have spacers of extrachromosomal origin. Microbiology.

[9] Barrangou R (2007). CRISPR provides acquired resistance against viruses in prokaryotes. Science.

[10] Brouns SJJ (2008). Small CRISPR RNAs guide antiviral defense in prokaryotes. Science.

[11] Gasiunas G, Barrangou R, Horvath P, Siksnys V (2012). Cas9-crRNA ribonucleoprotein complex mediates specific DNA cleavage for adaptive immunity in bacteria. Proc Natl Acad Sci USA.

[12] Jinek M (2012). A programmable dual-RNA-guided DNA endonuclease in adaptive bacterial immunity. Science.

[13] Cong L (2013). Multiplex genome engineering using CRISPR/Cas systems. Science.

[14] Cheng AW (2013). Multiplexed activation of endogenous genes by CRISPR-on, an RNA-guided transcriptional activator system. Cell Res.

[15] Chen B (2013). Dynamic imaging of genomic loci in living human cells by an optimized CRISPR/Cas system. Cell.

[16] Wu Y (2013). Correction of a genetic disease in mouse via use of CRISPR-Cas9. Cell Stem Cell.

[17] Wang D (2015). Adenovirus-mediated somatic genome editing of Pten by CRISPR/Cas9 in mouse liver in spite of Cas9-specific immune responses. Hum Gene Ther.

[18] Niu Y (2014). Generation of gene-modified cynomolgus monkey via Cas9/RNA-mediated gene targeting in one-cell embryos. Cell.

[19] Liang P (2015). CRISPR/Cas9-mediated gene editing in human tripronuclear zygotes. Protein Cell.

[20] Liu XS (2016). Editing DNA methylation in the mammalian genome. Cell.

[21] Stadtmauer EA (2020). CRISPR-engineered T cells in patients with refractory cancer. Science.

[22] Serganova I (2017). Enhancement of PSMA-directed car adoptive immunotherapy by PD-1/PD-L1 blockade. Mol Ther Oncolytics.

[23] Ren J (2017). Multiplex genome editing to generate universal CAR T cells resistant to PD1 inhibition. Clin Cancer Res.

[24] Nishida K (2016). Targeted nucleotide editing using hybrid prokaryotic and vertebrate adaptive immune systems. Science.

[25] Komor AC, Kim YB, Packer MS, Zuris JA, Liu DR (2016). Programmable editing of a target base in genomic DNA without double-stranded DNA cleavage. Nature.

[26] Koblan LW (2021). In vivo base editing rescues Hutchinson-Gilford progeria syndrome in mice. Nature.

[27] Chu SH (2021). Conversion of HbS to Hb G-makassar by adenine base editing is compatible with normal hemoglobin function. Blood.

[28] Rupp LJ (2017). CRISPR/Cas9-mediated PD-1 disruption enhances anti-tumor efficacy of human chimeric antigen receptor T cells. Sci Rep.

[29] Cox DBT (2017). RNA editing with CRISPR-Cas13. Science.

[30] Myhrvold C (2018). Field-deployable viral diagnostics using CRISPR-Cas13. Science.

[31] Anzalone AV (2019). Search-and-replace genome editing without double-strand breaks or donor DNA. Nature.

[32] Gao J, Luo T, Lin N, Zhang S, Wang J (2020). A new tool for CRISPR-Cas13a-based cancer gene therapy. Mol Ther Oncolytics.

[33] Dash PK (2019). Sequential LASER ART and CRISPR treatments eliminate HIV-1 in a subset of infected humanized mice. Nat Commun.

[34] Strecker J (2019). RNA-guided DNA insertion with CRISPR-associated transposases. Science.

[35] Martinez-Lage M (2020). In vivo CRISPR/Cas9 targeting of fusion oncogenes for selective elimination of cancer cells. Nat Commun.

[36] Fozouni P (2020). Direct detection of SARS-CoV-2 using CRISPR-Cas13a and a mobile phone. MedRxiv.

[37] Doudna JA, Charpentier E (2014). Genome editing. The new frontier of genome engineering with CRISPR-Cas9. Science.

[38] Amitai G, Sorek R (2016). CRISPR-Cas adaptation: insights into the mechanism of action. Nat Rev Microbiol.

[39] Marraffini LA, Sontheimer EJ (2008). CRISPR interference limits horizontal gene transfer in staphylococci by targeting DNA. Science.

[40] Hale CR (2009). RNA-guided RNA cleavage by a CRISPR RNA-Cas protein complex. Cell.

[41] Garneau JE (2010). The CRISPR/Cas bacterial immune system cleaves bacteriophage and plasmid DNA. Nature.

[42] Wiedenheft B, Sternberg SH, Doudna JA (2012). RNA-guided genetic silencing systems in bacteria and archaea. Nature.

[43] van der Oost J, Westra ER, Jackson RN, Wiedenheft B (2014). Unravelling the structural and mechanistic basis of CRISPR-Cas systems. Nat Rev Microbiol.

[44] Jiang F, Doudna JA (2015). The structural biology of CRISPR-Cas systems. Curr Opin Struct Biol.

[45] Deveau H (2008). Phage response to CRISPR-encoded resistance in *Streptococcus thermophilus*. J Bacteriol.

[46] Horvath P (2008). Diversity, activity, and evolution of CRISPR loci in *Streptococcus thermophilus*. J Bacteriol.

[47] Mojica FJM, Díez-Villaseñor C, García-Martínez J, Almendros C (2009). Short motif sequences determine the targets of the prokaryotic CRISPR defence system. Microbiology.

[48] Jiang F, Doudna JA (2017). CRISPR-Cas9 structures and mechanisms. Annu Rev Biophys.

[49] Jinek M (2014). Structures of Cas9 endonucleases reveal RNA-mediated conformational activation. Science.

[50] Nishimasu H (2014). Crystal structure of Cas9 in complex with guide RNA and target DNA. Cell.

[51] Anders C, Niewoehner O, Duerst A, Jinek M (2014). Structural basis of PAM-dependent target DNA recognition by the Cas9 endonuclease. Nature.

[52] Sternberg SH, Redding S, Jinek M, Greene EC, Doudna JA (2014). DNA interrogation by the CRISPR RNA-guided endonuclease Cas9. Nature.

[53] Hsu PD, Lander ES, Zhang F (2014). Development and applications of CRISPR-Cas9 for genome engineering. Cell.

[54] Lander ES (2016). The heroes of CRISPR. Cell.

[55] Makarova KS (2015). An updated evolutionary classification of CRISPR-Cas systems. Nat Rev Microbiol.

[56] Semenova E (2011). Interference by clustered regularly interspaced short palindromic repeat (CRISPR) RNA is governed by a seed sequence. Proc Natl Acad Sci USA.

[57] Wiedenheft B (2011). RNA-guided complex from a bacterial immune system enhances target recognition through seed sequence interactions. Proc Natl Acad Sci USA.

[58] Pattanayak V (2013). High-throughput profiling of off-target DNA cleavage reveals RNA-programmed Cas9 nuclease specificity. Nat Biotechnol.

[59] Jinek M (2013). RNA-programmed genome editing in human cells. Elife.

[60] Mali P (2013). RNA-guided human genome engineering via Cas9. Science.

[61] Knight SC (2015). Dynamics of CRISPR-Cas9 genome interrogation in living cells. Science.

[62] Ma H (2016). CRISPR-Cas9 nuclear dynamics and target recognition in living cells. J Cell Biol.

[63] Szczelkun MD (2014). Direct observation of R-loop formation by single RNA-guided Cas9 and Cascade effector complexes. Proc Natl Acad Sci USA.

[64] Vassylyev DG (2002). Crystal structure of a bacterial RNA polymerase holoenzyme at 2.6 A resolution. Nature.

[65] Plaschka C (2015). Architecture of the RNA polymerase II-mediator core initiation complex. Nature.

[66] Wu X (2014). Genome-wide binding of the CRISPR endonuclease Cas9 in mammalian cells. Nat Biotechnol.

[67] O’Geen H, Henry IM, Bhakta MS, Meckler JF, Segal DJ (2015). A genome-wide analysis of Cas9 binding specificity using ChIP-seq and targeted sequence capture. Nucleic Acids Res.

[68] Kuscu C, Arslan S, Singh R, Thorpe J, Adli M (2014). Genome-wide analysis reveals characteristics of off-target sites bound by the Cas9 endonuclease. Nat Biotechnol.

[69] Josephs EA (2015). Structure and specificity of the RNA-guided endonuclease Cas9 during DNA interrogation, target binding and cleavage. Nucleic Acids Res.

[70] Jiang F (2016). Structures of a CRISPR-Cas9 R-loop complex primed for DNA cleavage. Science.

[71] Doudna JA (2020). The promise and challenge of therapeutic genome editing. Nature.

[72] Ghosh D, Venkataramani P, Nandi S, Bhattacharjee S (2019). CRISPR-Cas9 a boon or bane: the bumpy road ahead to cancer therapeutics. Cancer Cell Int.

[73] Rafii S, Tashkandi E, Bukhari N, Al-Shamsi HO (2022). Current status of CRISPR/Cas9 application in clinical cancer research: opportunities and challenges. Cancers.

[74] Rosenblum D (2020). CRISPR-Cas9 genome editing using targeted lipid nanoparticles for cancer therapy. Sci Adv.

[75] Razeghian E (2021). A deep insight into CRISPR/Cas9 application in CAR-T cell-based tumor immunotherapies. Stem Cell Res Ther.

[76] Guo X (2018). Disruption of PD-1 enhanced the anti-tumor activity of chimeric antigen receptor T cells against hepatocellular carcinoma. Front Pharmacol.

[77] Azangou-Khyavy M (2020). CRISPR/Cas: from tumor gene editing to T cell-based immunotherapy of cancer. Front Immunol.

[78] Lu Y (2020). Safety and feasibility of CRISPR-edited T cells in patients with refractory non-small-cell lung cancer. Nat Med.

[79] Xu L (2019). CRISPR-edited stem cells in a patient with HIV and acute lymphocytic leukemia. N Engl J Med.

[80] Uddin F, Rudin CM, Sen T (2020). CRISPR gene therapy: applications, limitations, and implications for the future. Front Oncol.

[81] Naeem M, Majeed S, Hoque MZ, Ahmad I (2020). Latest developed strategies to minimize the off-target effects in CRISPR-Cas-mediated genome editing. Cells.

[82] Gleditzsch D (2018). PAM identification by CRISPR-Cas effector complexes: diversified mechanisms and structures. RNA Biol.

[83] Le Rhun A, Escalera-Maurer A, Bratovič M, Charpentier E (2019). CRISPR-Cas in Streptococcus pyogenes. RNA Biol.

[84] Lino CA, Harper JC, Carney JP, Timlin JA (2018). Delivering CRISPR: a review of the challenges and approaches. Drug Deliv.

[85] Ihry RJ (2018). p53 inhibits CRISPR-Cas9 engineering in human pluripotent stem cells. Nat Med.

[86] Cullot G (2019). CRISPR-Cas9 genome editing induces megabase-scale chromosomal truncations. Nat Commun.

[87] Li H (2020). Applications of genome editing technology in the targeted therapy of human diseases: mechanisms, advances and prospects. Signal Transduct Target Ther.

[88] Charlesworth CT (2019). Identification of preexisting adaptive immunity to Cas9 proteins in humans. Nat Med.

[89] Moreno AM (2019). immune-orthogonal orthologues of AAV capsids and of Cas9 circumvent the immune response to the administration of gene therapy. Nat Biomed Eng.

[90] Adli M (2018). The CRISPR tool kit for genome editing and beyond. Nat Commun.

[91] Kleinstiver BP (2015). Engineered CRISPR-Cas9 nucleases with altered PAM specificities. Nature.

[92] Kleinstiver BP (2016). High-fidelity CRISPR-Cas9 nucleases with no detectable genome-wide off-target effects. Nature.

[93] Deltcheva E, Chylinski K, Sharma CM (2011). CRISPR RNA maturation by trans-encoded small RNA and host factor RNase III. Nature.

[94] Zetsche B, Gootenberg JS, Abudayyeh OO (2015). Cpf1 is a single RNA-guided endonuclease of a class 2 CRISPR-Cas system. Cell.

